# Adverse Effects of the Long-term use of an N95 Respirator in Healthcare Workers

**DOI:** 10.14789/ejmj.JMJ24-0050-OA

**Published:** 2025-04-18

**Authors:** HIROKI TAKAMI, TAKASHI MITSUHASHI, DAISUKE USUDA, TOMOHISA NOMURA, MANABU SUGITA

**Affiliations:** 1 Department of Emergency and Critical Care Medicine, Juntendo University Nerima Hospital, Tokyo, Japan; 1 Department of Emergency and Critical Care Medicine, Juntendo University Nerima Hospital, Tokyo, Japan; 2 Department of Neurosurgery, Juntendo University Nerima Hospital, Tokyo, Japan; 2 Department of Neurosurgery, Juntendo University Nerima Hospital, Tokyo, Japan

**Keywords:** N95 respirator, adverse effects, healthcare worker, work efficiency, COVID-19

## Abstract

**Objectives:**

Healthcare workers must take stringent infection control measures against coronavirus disease. Previous reports have indicated that N95 respirators cause fatigue, discomfort, and physical symptoms, such as headaches. We aimed to comparatively analyze the effect of the use of surgical and N95 respirators for long hours on the performance of healthcare workers. This is the first study to validate the effect of wearing N95 respirators on work efficiency.

**Materials and Methods:**

This study was conducted from April 2021 to October 2021 during the COVID-19 pandemic in Japan. Healthy healthcare workers at an emergency department were subjected to a performance task program comprising four tasks conducted before and after wearing a surgical mask/N95 respirator for at least 4 h, and the results were compared.

**Results:**

The study included 17 (male, 8 [47.1%]) healthcare workers. The age ranged from 22 to 32 (mean, 26.6) years. For each task, the rate of change in the percentage of correct responses, rate of decline in reaction time, and rate of decline in reaction time for correct responses were calculated before and after wearing the two types of masks. There was no statistically significant difference in the rate of decline in reaction time between the masks for all tasks. However, there was a trend toward a high rate of decline in the alphanumeric detection task.

**Conclusions:**

Using an N95 respirator for 4 h continuously did not adversely affect work efficiency. Although prolonged work under conditions of discomfort should be avoided, it is unlikely that N95 respirators will adversely affect the performance of healthcare workers.

## Introduction

Due to the significant increase in the number of patients with COVID-19 during the global pandemic, healthcare workers, especially those working in emergency and outpatient departments, were required to wear personal protective equipment (PPE) such as N95 respirators more frequently than other healthcare workers to ensure infection prevention. The “N” in N95 stands for “Not resistant to oil” which is denoted by NIOSH (National Institute for Occupational Safety and Health), and the “95” indicates particle filtration efficiency; an N95 respirator can prevent the inhalation of 95% of all airborne particles, including very small particles. On the other hand, surgical masks provide a barrier against large respiratory particles. However, they do not prevent leakage around the mask during inhalation because of poor adherence to the skin. Thus, surgical masks do not effectively protect healthcare workers against infection when directly caring for patients with COVID-19.

Previous studies on the N95 respirator have reported that prolonged wearing causes discomfort and headache^1, 2)^. The level of discomfort increases over time, regardless of the type of respirator^3)^. In addition, studies have reported direct physical effects of wearing N95 respirators, such as increased heart rate, body temperature, and humidity^4)^. The tight seal of N95 respirators increases the blood's carbon dioxide (CO_2_) concentration, causing hypercapnia, which leads to cognitive decline^5)^.

The impaired ventilation, cardiopulmonary capacity, and comfort of N95 respirators compared with surgical masks has led to concerns about healthcare workers developing health problems from prolonged work under such conditions, as well as the danger to patients who receive medical treatment from workers wearing N95 respirators^6)^. Error in judgment in treating patients with severe conditions can make a difference between life and death. This is an important issue from the standpoint of patient safety.

To date, no report has examined the effects of the prolonged use of N95 respirators on work efficiency. However, wearing N95 respirators for extended periods while experiencing discomfort or headaches may cause a decrease in work efficiency. Therefore, we hypothesized that healthcare workers' work efficiency might decrease when wearing N95 respirators for extended periods. We compared healthcare workers' performance in the emergency department before and after wearing N95 respirators and surgical masks to determine if any adverse effects were associated with using an N95 respirator. This is the first study to validate the effect of wearing N95 respirators on work efficiency.

## Materials and Methods

### Study design

This study was conducted at Juntendo University Nerima Hospital from April 2021 to October 2021 during the COVID-19 pandemic in Japan. We recruited healthy participants consisting of emergency room healthcare professionals with no prior history of illness, including underlying respiratory diseases. Both oral and written informed consent were obtained from all the participants. The participants were alternately assigned to wear a surgical mask on day 1 and an N95 respirator on day 2 or vice versa. When wearing an N95 respirator, the fit task was always conducted. The participants were instructed not to remove their masks during the study period. All experiments were conducted during the day shift. This study was approved by the Ethical Committee of Juntendo University Nerima Hospital and was conducted in accordance with the latest revision of the Declaration of Helsinki.

### Evaluation of work efficiency

We chose to measure simple arithmetic ability and reaction time to assess the effect of prolonged use of N95 respirators on work efficiency. Whether a decrease in cognitive function tasks is directly related to a decrease in work efficiency is thought to depend on the specific work content and situation. Therefore, since it is difficult to accurately predict the effect on actual work, we thought that the effect could be evaluated as accurately as possible by conducting the task in an actual work environment.

Therefore, we decided to evaluate four tasks: (1) a simple task: measuring reaction speed to presented marks, (2) an addition task: adding presented numbers, (3) an alphabet-number recognition task: measuring reaction speed to recognize target letters (alphabet and numbers), and (4) a short-term memory task: judging multiple numbers of target letters. The following four tasks have been evaluated. The integrated software, Performance Test Program (PTP) N-020-10000 (Norpro Light Systems, Tokyo, Japan), was used for these tasks (http://www.norupro.ne.jp/pdf/P01.pdf). Four tasks were administered and the percentage change in percent correct response, percent decrease in reaction time, and percent decrease in correct reaction time were calculated before and after wearing the two mask types.

Participants wore surgical masks and performed all four tasks once. At a later date, they wore N95 respirators and similar tasks once. These tasks were administered before and after a 4-hour normal working day, wearing one of the masks. On each occasion, participants were also asked about symptoms such as headache and dyspnea.

For these four tasks, the learning effect intervention was minimized by having the participants practice thoroughly before administering the task.

Regarding the wearing of surgical masks, N95 respirators were worn only when responding to patients with fever or symptoms suggestive of COVID-19. Participants used blue surgical masks (White Cross Corporation, Tokyo, Japan) and 3M™ AURA™ N95 1870 + MED respirators (3M Company, Saint Paul, MN, USA).

### Statistical analysis

Stata 16.1 package (StataCorp, College Station, TX, USA) was used to perform all statistical analyses. The normality of the distribution of continuous variables was tested using the D'Agostino-Pearson method. T-tests were used to assess statistical differences between surgical and N95 respirators. *P*-values were reported to determine statistical significance. Statistical significance was set at a two-sided *p*-value of <0.05.

## Results

In all, 17 (male, 8 [47.1%]) participants were included. The industry of the participants included 3 emergency physicians and 14 nurses. The age ranged from 22 to 32 (mean, 26.6) years. There was no smoker among the participants. In addition, no participant removed mask during observation period. No participant complained of headaches or other symptoms requiring medical treatment after wearing each mask for 4 h. All participants complained of subjective symptoms of discomfort and breathlessness while wearing an N95 respirator, although none stopped wearing the mask. During the study period, the average number of patients who visited the emergency room was 1473 per month and transported by ambulance was 531 per month.

**Figure 1:**
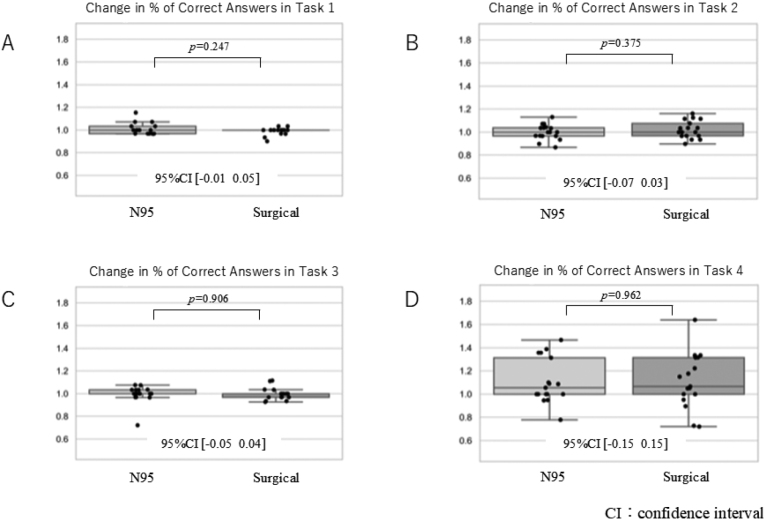
Change in % of correct answers In each graph, values >1 indicate the rate of increase before and after wearing the mask, whereas values <1 indicate the rate of decrease before and after wearing the mask.

**Figure 2:**
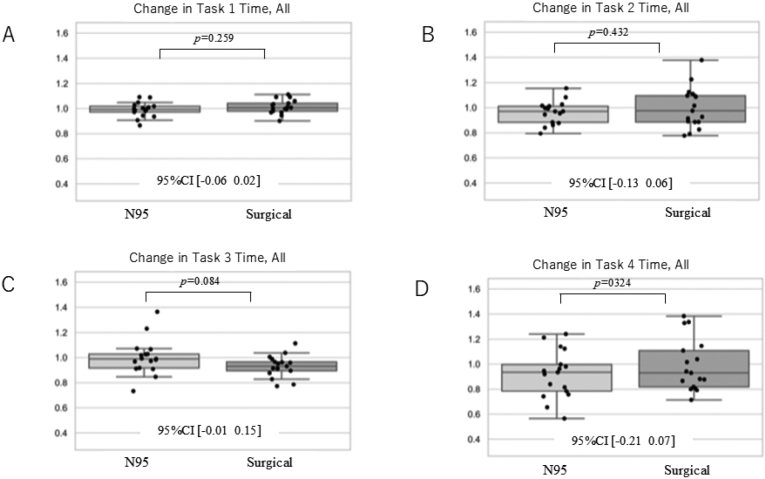
Δtime all – Reaction time related to all answers In each graph, values >1 indicate the rate of increase before and after wearing the mask, whereas values <1 indicate the rate of decrease before and after wearing the mask.

**Figure 3:**
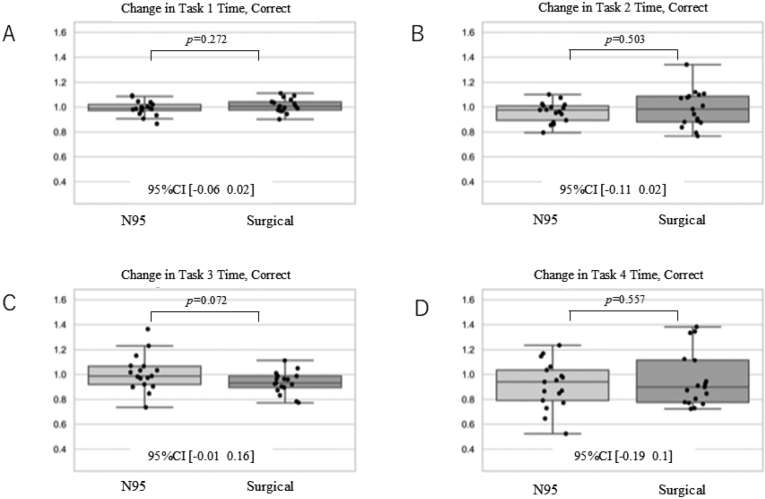
Δtime correct - Reaction time restricted to correct answers only In each graph, values >1 indicate the rate of increase before and after wearing the mask, whereas values <1 indicate the rate of decrease before and after wearing the mask.

According to the D'Agostino-Pearson test, all results obtained as continuous variables followed the normal distribution. Figure 1 shows a change in % of correct answers. As a result, there was no difference in the change in the percentage of correct answers on all tasks. Figure 2 shows Δtime all, namely reaction time related to all answers. The time to reply to all questions that did not involve correct or incorrect answers showed no significant difference for all tasks. However, in task 3, those wearing the N95 respirator tended to take longer to answer the questions before and after wearing the mask. Figure 3 shows Δtime correct, namely the reaction time restricted to correct answers only. A comparison of the change in time for correctly answered questions showed no significant differences in all tasks. Those wearing the N95 respirator took longer to answer the questions in task 3, both before and after wearing the mask. As summary, there was no significant difference in the percent change in percent correct response between the two mask types for all tasks. In addition, there was no statistically significant difference in the rate of decrease in reaction time for any of the tasks. On the other hand, there was a trend towards a higher rate of decrease in performance on task 3.

## Discussion

Our study shows that an N95 respirator can continuously be worn for up to 4 h without problems. Although prolonged work in an environment that causes discomfort should be avoided, using this mask is unlikely to affect medical care directly. In previous reports, even though using N95 respirators was effective as an infection control measure, headaches, dryness of the eyes and nose, and acne were pointed out as the adverse effects of long periods of mask use^2, 5)^. In another study, using N95 respirators significantly increased the occurrence of headache, dyspnea, drowsiness, and numbness compared with surgical masks, leading to decreased alertness and concentration, as assessed by a questionnaire survey^7)^. A treadmill experiment using an N95 respirator resulted in increased heart and respiratory rates and a significant increase in transcutaneous partial pressure of CO_2_, although blood oxygen levels remained unchanged^8)^. It has also been reported that wearing an N95 respirator significantly reduces minute ventilation volumes^6)^. Decreased minute ventilation volume and increased transcutaneous carbon dioxide concentrations indicate elevated blood CO_2_ levels. Elevated CO_2_ concentrations have been reported to alter intracranial arterial hemodynamics, causing discomfort, fatigue, dizziness, headache, shortness of breath, general malaise, lethargy and drowsiness, and even cognitive decline^5, 9-12)^.

A recent study showed that the quality of CPR (cardiopulmonary resuscitation) was reduced by fatigue when using an N95 respirator compared to a surgical mask^13)^. While previous studies have suggested that wearing N95 respirators may impair performance, this study found no negative effect of mask use on work performance. In this study, there was a learning effect with improvements in reaction time and percentage correct before and after wearing the mask in each task, but only the rate of decrease in reaction time in task 3 was higher for the surgical mask than for the N95 respirator. Although it is possible that surgical masks may have less of an impact on work performance than N95 respirators when viewed from this perspective, the current validation did not find a significant change in performance with N95 respirator wear, either in correct response rate or reaction time.

Reports have shown that when healthy participants wore a surgical mask for one hour of light to moderate exercise, heart rate, respiratory rate, and transcutaneous carbon dioxide concentration increased^14)^. In addition, the use of a surgical mask during 30 minutes of continuous exercise has been reported to be associated with decreased minute ventilation, increased heart rate, and prolonged inspiratory time^15)^.

A report examining the effects of mask use in professional chess players showed that mask use decreased cognitive function^16)^.

It is possible that healthcare workers who wore surgical masks for routine infection prevention prior to the COVID-19 outbreak were accustomed to the burden of wearing surgical masks; Su et al. also found that healthcare workers who wore N95 respirators and surgical masks during an 8-hour shift in the emergency department reported no adverse physiological or psychological symptoms^17)^.

This study focused on the effects of wearing N95 respirators at work. However, in actual operations, patients with infectious diseases are handled by workers wearing full PPE, including gowns, eye guards, and masks. This study focused on the effectiveness of wearing N95 respirators in the workplace. However, the comparison of standard precautions to full PPE may have influenced the results, because in the real world, workers wearing full PPE, including gowns, eye protection, and masks, handle patients with infectious diseases.

Healthcare workers who handle critically ill patients in emergency departments and intensive care units need to be alert to the possibility of a deteriorating work environment, as a single mistake can directly affect a patient's life. This study was conducted in a hospital setting, suggesting that the wearing of N95 respirators is unlikely to interfere with medical practice. However, given that medical masks have also been reported to increase discomfort due to perspiration and humidity, there is concern that prehospital activities (e.g., emergency medical teams, ambulances, and disaster relief efforts) may be affected by environmental factors such as temperature and humidity, and thus the duration of wear should be considered.

This study had some limitations. First, this was a single-center study, and the number of participants was small. In addition, the task was conducted 1 year after the COVID-19 epidemic, which may have caused its adaptability. Second, the busyness of the emergency department varies from day to day, resulting in workload variations. We could not uniform the workload of busyness. Third, factors related to performance including work content, fatigue level, and other information, which might have affected the results were not evaluated. The results could have been different if the task had been limited to more intensive situations, such as lengthy surgeries or dealing with patients in cardiopulmonary arrest. Fourth, we only validated 4 hours workload effects, and we need to make more verifications including wearing N95 respirators longer. Therefore, we are unable to discuss the relationship between the length of N95 respirator and the effect on their actual work. Fifth, we didn't consider the cause, follow up, or care for symptoms that occurred during tasks. Therefore, we are unable to discuss the relationship between wearing N95 respirators and the symptoms.

## Conclusion

This study is the first report to assess the effect of wearing N95 respirators on work efficiency. Our study showed that up to 4 h of continuous wearing of an N95 respirator might not hinder the performance of healthcare workers. Although prolonged work in an environment that causes discomfort at work should be avoided, mask use is unlikely to have a direct adverse effect on medical care. In the future, work efficiency using masks should be evaluated in different situations of high workloads, such as long surgeries and life-saving care for critically ill patients.

## Funding

This research received no external funding.

## Author contributions

Concept and design: HT, TM, MS. Acquisition, analysis, or interpretation of data: HT, TM, DU. Drafting of the manuscript: HT, TM, DU, TN. Critical review of the manuscript for important intellectual content: TM, DU, TN, MS. Supervision: TM, MS. All authors have reviewed the final version to be published and agreed to be accountable for all aspects of the work.

## Conflicts of interest statement

The authors declare that they have no competing interests to disclose.
